# A Cross-Sectional Study of Neutrophil-to-Lymphocyte Ratio in Diagnosing Acute Appendicitis in Hospital Melaka

**DOI:** 10.21315/mjms2019.26.6.6

**Published:** 2019-12-30

**Authors:** Khairol Ashraf Ahmad, Noorharisman Ideris, Syed Hassan Syed Abd Aziz

**Affiliations:** 1 Department of Surgery, School of Medical Sciences, Universiti Sains Malaysia, Kubang Kerian, Kelantan, Malaysia; 2 Department of Surgery, Hospital Melaka, Jalan Mufti Haji Khalil, Melaka, Malaysia

**Keywords:** NLR, neutrophil-to-lymphocyte ratio, neutrophil, lymphocyte, acute appendicitis, perforated appendicitis, appendectomy, negative appendicectomy, white appendix

## Abstract

**Background:**

Acute appendicitis is one of the most common surgical emergencies. However, its proper diagnosis is complicated. This study aims to evaluate the ability of the neutrophil-to-lymphocyte ratio (NLR) to diagnose acute appendicitis in pre-operative state.

**Methods:**

Histopathological examination of appendicectomies conducted between 2016 and 2017 in Melaka Hospital, Malaysia were traced and categorised into three groups: i) G1 (normal appendix), ii) G2 (acute appendicitis) and iii) G3 (perforated appendicitis). The reports were randomised and a total of 338 samples were collected. NLR values were compared between the three different groups and analysed.

**Results:**

The median values of NLR for G1, G2 and G3 were 2.37, 5.25 and 9.27, respectively. We found a statistically significant difference in NLR between G1 and G2 (*P* < 0.001), and G2 and G3 (*P* < 0.001). The diagnostic values of NLR for acute appendicitis and perforated appendicitis were 3.11 (sensitivity: 75.23%, specificity: 68.70%) and 6.17 (sensitivity: 76.32%, specificity: 58.72%), respectively. There was a substantial correlation between NLR and disease severity, and a moderate correlation between NLR and duration of admission.

**Conclusion:**

NLR, with a sensitivity of 75.23% and specificity of 68.70%, is a useful and reliable adjunct in diagnosing acute appendicitis. Hence, it will help in reducing the rate of negative appendicectomies.

## Introduction

Acute appendicitis is one of the most common causes of acute abdomen and is the most frequent condition leading to emergent abdominal surgeries. Patients with acute appendicitis are commonly presented with symptoms of pain in the right lower abdomen, nausea, vomiting and anorexia. However, about 40% of people do not have these typical symptoms (1). If undetected, acute appendicitis can progress to perforated appendicitis, giving rise to severe complications like painful inflammation of the inner lining of the abdominal wall (peritonitis) and sepsis.

Acute appendicitis affects 1.5–1.9 individuals in a population of 100,000 and is 1.4 times more common in men (2). The lifetime risk of suffering from acute appendicitis is 7%, with perforation rates being 17%–20% (2). The mortality risk of this condition is less than 1% in the general population but can rise to 50% among the elderly population (2). It was reported that the mortality rate from acute appendicitis in Malaysia is 4 per 10,000 population in the year 2013 (3).

Diagnosing acute appendicitis is a challenging task because many clinicians rely on the signs and symptoms presented by the patient. In case of elusive diagnosis, close observation, laboratory tests, and imaging can be helpful. However, the accurate diagnosis of acute right iliac fossa pain remains a challenging clinical problem as the differential diagnosis of pain in this region is not straight-forward.

To overcome morbidity and mortality of perforation before surgery, a negative appendicectomy is somewhat acceptable traditionally. However, in recent years, many have considered this unacceptable since the surgical procedure itself could be a potential cause of morbidity and mortality. Although appendicectomy has markedly reduced the morbidity, it has led to an increase in diagnostic error rate (4).

The rate of negative appendicectomy remains high, varying between 15%–30% globally (5). The University Malaya Medical Centre, Malaysia reported that the negative appendicectomy rate is as high as 19.3% (4).

Negative appendicectomy rates can be reduced with an accurate and prompt diagnosis. There is a number of useful diagnostic modalities for acute appendicitis, including evaluation of clinical symptoms, scoring systems such as Alvarado and RIPASA score, and imaging methods such as ultrasound and computed tomography (CT) scan. Scoring systems provide an objective means of predicting acute appendicitis; however, they lack sensitivity and specificity and provide no insight as to how advanced is the inflammatory process (5). Although CT scan can reduce the rate of negative appendicectomy from 24% to 7.6% (2), such valuable tools are expensive and are not ubiquitously available in many centres.

Therefore, the use of simple diagnostic tools in diagnosing acute appendicitis and reducing the overall rate of negative appendicectomy is of great importance, especially if they are cheap, easy to use and easy to interpret. Basic blood investigation, such as full blood count (FBC), is undertaken as a baseline investigation in all the patients presented with right iliac fossa pain and can be used as a diagnostic tool to detect acute appendicitis. FBC is cheap and readily available in medical centres and can detect haemoglobin levels, total leucocyte count and platelet count, along with differential counts for the patient. Although patients with acute appendicitis can also be presented with leucocytosis, its specificity is low due to a myriad of abdominal conditions associated with leucocytosis (5). Several studies have also reported that leucocytosis can be very non-specific at times.

Thus, the usage of neutrophil-to-lymphocyte ratio (NLR) that can be derived from the differential count, can act as an adjunct in diagnosing acute appendicitis in general and reduce the rate of negative appendicectomy.

Our study aims to investigate the usefulness of the NLR as a diagnostic tool in detecting acute appendicitis such that it can be included as a routine practise in hospital settings. Since there is quite a number of patients admitted with suspected acute appendicitis and subjected for appendicectomy, like in our hospital, NLR will be helpful in reducing the rate of negative appendicectomy and prevent unnecessary morbidity caused by the procedure itself. In addition, there are no previous reports co-relating NLR and acute appendicitis in the Malaysian population.

## Methods

Sample size calculation for this study was conducted using G*Power software version 3.1 by using statistical power of *F*-tests and ANOVA. The significant level (α) was set at 0.05, the power (1-β) was 0.8 and the number of groups was three. The effect size (f) was 0.174. Thus, the total sample size calculated was 324. Additional 10% sample size was required for considering estimated 10% missing data. So, the total sample size needed was 357.

A total of 1,597 patients underwent appendicectomy for suspected acute appendicitis during this period of study. The histopathology examination (HPE) of patient’s appendix was traced and grouped into Group 1, Group 2 and Group 3. A total of 119 samples were randomised equally for each group by using ‘Research Randomizer’ application from Randomizer website (www.randomizer.org). Medical records of the selected HPE report were reviewed. Patients younger than the age of 12 years, pregnant patients, patients who underwent appendicectomy during surgery for other indications and patients with incomplete medical records were excluded from this study.

Age, gender, ethnicity, date of admission and discharge from hospital, date of appendicectomy, total white blood cells (TWBC) count, lymphocytes count, neutrophils count and platelet count of the patients were recorded in our data collection form. The NLR values were then calculated.

### Statistical Analysis

Data were analysed using SPSS version 24. Sensitivity and specificity were analysed using STATA version 14. There were no significant outliers that warrant data removal, as assessed by inspecting boxplots. Descriptive statistics were used to summarise the socio-demographic characteristics of the subjects. Numerical data were presented as mean (SD), median (IQR) and range. Duration of admission and NLR did not follow normal distribution. Categorical data were represented as frequencies (percentage). Numerical variables were compared across three groups with ANOVA. If the data was not normally distributed, Kruskal-Wallis test was applied as an alternative. Categorical data were compared across three groups with Pearson’s chi-squared test. If the assumption of Pearson’s chi-squared test was not fulfilled, Fisher’s exact test was employed.

The differences between the groups were compared by Kruskal-Wallis test since NLR was not normally distributed. Meanwhile, categorical data were analysed by Pearson’s chi-squared test or Fisher’s exact test. A value of *P* < 0.05 is considered statistically significant.

The diagnostic value of NLR was determined by receiver operating characteristic (ROC) analysis. The cut-off point of NLR in predicting the disease was selected based on the highest percentage of correct classifications. Sensitivity and specificity were also reported. Spearman correlation was then used to determine the correlation of NLR and duration of admission as well as disease severity.

## Results

Out of the 357 patient samples, 338 samples were considered in our study. Nineteen samples were excluded due to incomplete data. Majority of patients [324] underwent open appendicectomy, two patients underwent laparoscopic appendicectomy, two underwent laparoscopic converted to open appendicectomy and 10 underwent lower midline laparotomy appendicectomy.

The sociodemographic and clinical profiles of the study population are depicted in Table 1. There were 115 patients in Group 1, 109 patients in Group 2 and 114 patients in Group 3. The mean age of subjects was 26.61 years, 24.71 years and 26.36 years for Groups 1, 2 and 3, respectively. There was no significant difference in age between the three groups of subjects (*P* = 0.479). Most of the subjects in Group 1 were females (75.7%), Group 2 had equal proportions of males and females, while most of the subjects in Group 3 were male (72.8%). Pearson’s chi-squared test was conducted to determine if there were significant differences in gender between groups. There was a significant difference in gender proportion (male/female ratio) between the Groups 1 and 2 (*P* = 0.001), Groups 2 and 3 (*P* < 0.001), and Groups 1 and 3 (*P* < 0.001) as presented in Table 2. We found that most of the subjects in our study were Malays, i.e., 85.2% in Group 1, 90.8% in Group 2 and 78.1% in Group 3. The non-Malay constituted about 14.8% in Group 1, 9.2% in Group 2 and 21.9% in Group 3. There was a significant difference in ethnic origins between Group 2 and Group 3 (*P* = 0.009). However, no significant difference was observed between Groups 1 and 2 (*P* = 0.198) and between Groups 1 and 3 (*P* = 0.162), as shown in Table 3. In terms of the duration of admission in hospital, subjects in Group 1 and Group 2 were admitted for 2–7 days (median of 3 days) and 2–8 days (median of 3 days), respectively. The duration of admission for Group 3 was the longest, i.e., 2–21 days (median of 5 days). We observed a significant difference in the durations of admission between the three groups. The mean TWBC of the subjects was 9.73, 12.98, and 16.20 for Groups 1, 2 and 3, respectively and the differences were statistically significant (*P* < 0.001) between the three groups. The mean for lymphocytes count in Groups 1, 2 and 3 was 2.55, 2.04 and 1.64, respectively; the mean of neutrophils count was 6.47 (Group 1), 10.34 (Group 2) and 13.64 (Group 3). There was a statistically significant difference in the mean of lymphocytes and neutrophils count between the three groups (*P* < 0.001). Mean of platelet count in Groups 1, 2 and 3 was 308.14, 306.98 and 282.39, respectively. There was also a statistically significant difference in the mean of platelet count between the groups (*P* = 0.041).

**Figure 1:**
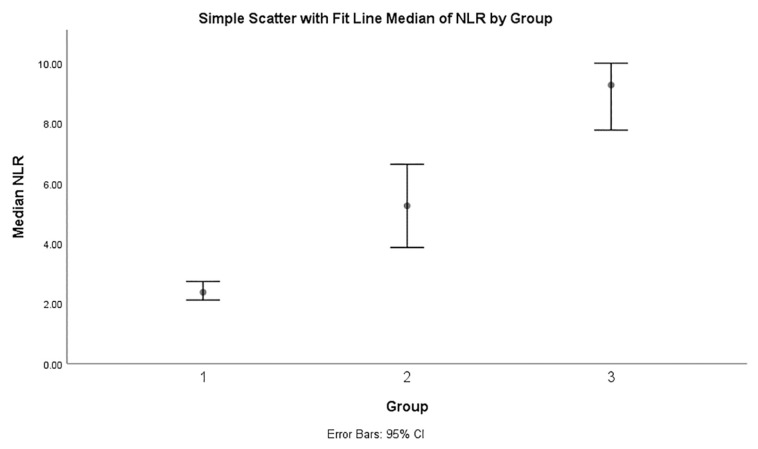
Comparison of median NLR between Group 1: normal appendix, Group 2: acute appendicitis and Group 3: perforated appendicitis

The NLR was calculated and compared between the three groups, as depicted in Table 4. For Groups 1, 2 and 3, the mean of NLR was 2.95, 6.66 and 10.66, respectively; meanwhile, the median of NLR was 2.37, 5.25, and 9.27 in Groups 1, 2 and 3, respectively. The median NLR was the highest in Group 3 (9.27; IQR: 7.07) compared to Group 1 (2.37; IQR: 1.84) and Group 2 (5.25; IQR: 6.81), which was a statistically significant difference (*P* < 0.001) (Figure 1). A Kruskal-Wallis test was then performed to determine if there were any differences in NLRs between the groups. Median of NLRs was significantly different between the three different groups (*P* < 0.001). Subsequently, pairwise comparisons were performed using Dunn’s procedure with a Bonferroni correction across multiple comparisons. Adjusted *P*-values are presented. This post hoc analysis revealed statistically significant differences in NLRs across all the pair-wise comparisons between Group 1 (2.37) and Group 2 (5.25) (*P* < 0.001), and Group 2 (5.25) and Group 3 (9.27) (*P* < 0.001) as well as Group 1 and Group 3 (*P* < 0.001).

**Figure 2:**
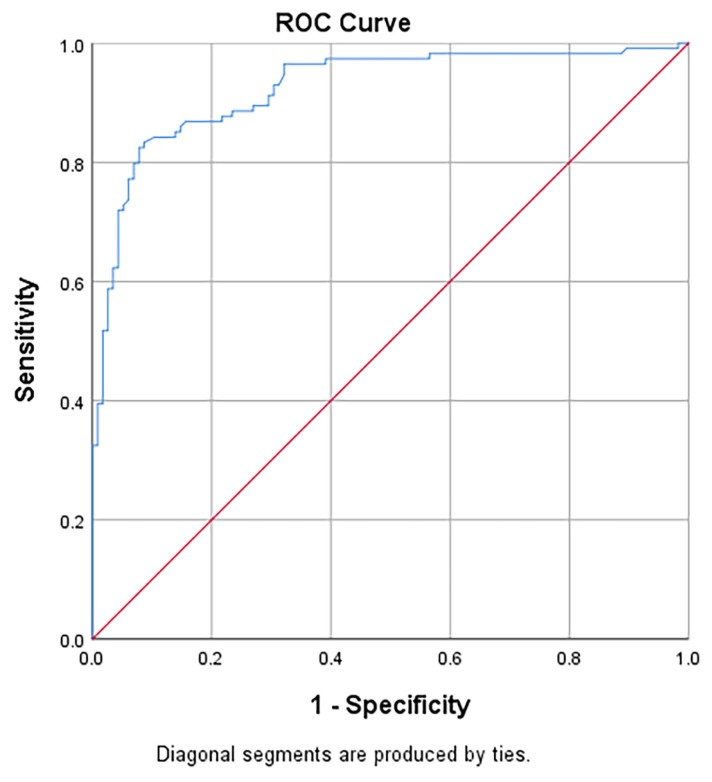
Area under the receiver operating characteristic curves (AUC) of NLR in predicting the diagnosis of Group 3: perforated appendicitis over Group 1: normal appendix

**Figure 3:**
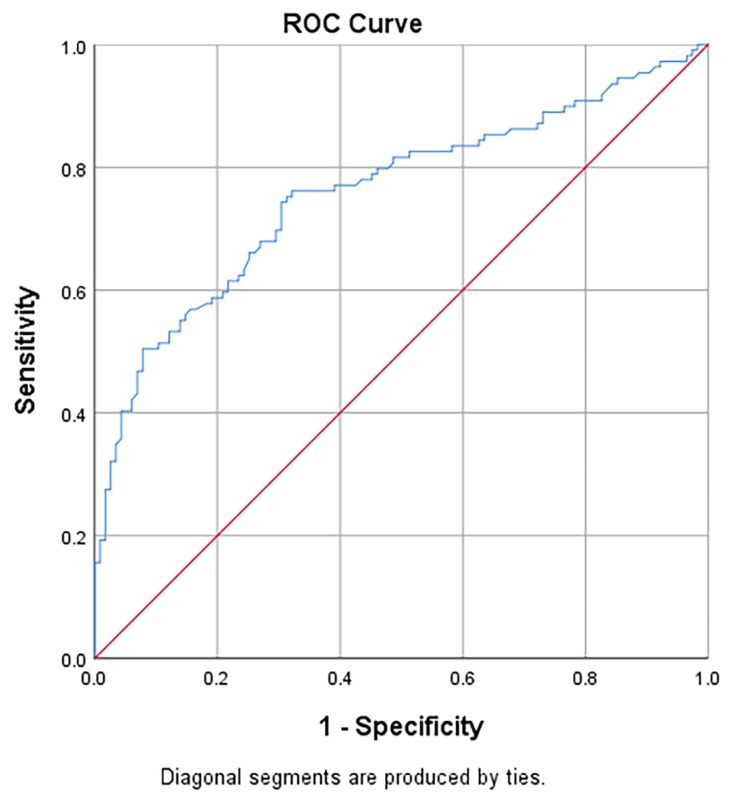
Area under the receiver operating characteristic curves (AUC) of NLR in predicting the diagnosis of Group 2: acute appendicitis over Group 1: normal appendix

**Figure 4:**
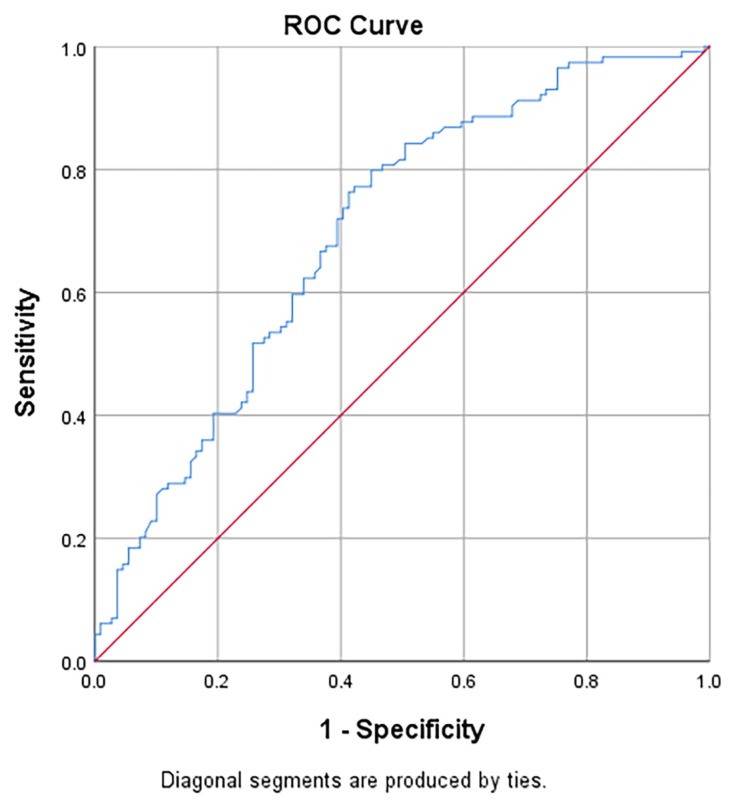
Area under the receiver operating characteristic curves (AUC) of NLR in predicting the diagnosis of Group 3: perforated appendicitis over Group 2: acute appendicitis

Table 5 presents the area under the receiver operating characteristic curves (AUC), sensitivity, specificity, positive likelihood ratios (LR+) and negative likelihood ratios (LR−) of NLR in predicting the diagnosis of acute appendicitis and perforated appendicitis. For Group 3 versus Group 1, the AUC of NLR was 0.84 (95% CI: 0.80, 0.89) and significantly different from the null hypothesis value of 0.5 (Figure 2). The sensitivity and specificity with cut-off point ≥ 5.11 were 83.3% and 91.3%, respectively. For Group 2 versus Group 1, the AUC of NLR was 0.76 (95% CI: 0.69, 0.82) and statistically different from the null hypothesis value of 0.5 (Figure 3). The sensitivity and specificity with cut-off point ≥ 3.11 were 75.2% and 68.7%, respectively. For Group 3 versus Group 2, the AUC of NLR was 0.70 (95% CI: 0.63, 0.77) and statistically different from the null hypothesis value of 0.5 (Figure 4). The sensitivity and specificity with cut-off point ≥ 6.17 were 76.3% and 58.7%, respectively.

Table 6 outlined the correlation between NLR and disease severity as well as the duration of admission. We observed a statistically significant, positive and substantial correlation between NLR and disease severity (*r* = 0.62; *P* < 0.001). Similarly, there was a statistically significant, positive but moderate correlation between NLR and duration of admission (*r* = 0.27; *P* < 0.001).

## Discussion

Acute appendicitis is one of the most common causes of acute abdominal pain in adults and also the paediatric age group and affects approximately 7% of the population (6). Diagnosis of acute appendicitis is quite a challenging task, especially in obese, females and young patients, where the diagnosis of appendicitis is largely based on patient’s signs and symptoms (7). Early detection is not always easy, and delays in diagnosis can cause morbidity and mortality (8). The decision to observe the patient until complete diagnosis or operate early to prevent unwanted complications such as perforation and peritonitis represents a serious dilemma for a surgeon (9). An early operation may result in the removal of the normal appendix and might contribute to unnecessary surgical procedures and morbidity of the patient (10). Ultrasound and CT imaging can be used in diagnosing appendicitis (11); however, not all hospitals, especially in a rural setting, are equipped with such imaging facilities. Furthermore, these imaging tools might not always help in achieving an accurate diagnosis (12). Thus, surgeons are still in need of an accurate, simple, inexpensive and easy diagnostic test in order to diagnose acute appendicitis, such as NLR (13).

Incidence of acute appendicitis and perforated appendicitis in our study was found to be higher among adolescents and young adults across all the three subject groups, where the median age was 20–23 years. In addition, there was no significant difference in age between the groups. The incidence was similarly reported in other studies (14, 15). Most of the patients diagnosed with perforated appendicitis group (Group 3) were males; however, the majority of normal appendix patients group (Group 1) were females. There was a statistically significant difference in gender proportions between the three patient groups. Majority of male subjects were presented with perforated appendicitis in our study; this may be because males tend to have higher threshold for pain compared to females, and most of them had a history of oral antibiotic medications. Thus, they presented late to the hospital with signs and symptoms of perforated appendicitis. As a result, male patients in our study tended towards severe forms of appendicitis such as perforated appendicitis,; this is in agreement with previous reports from Ishizuka et al. (16). On the other hand, HPE reported was a normal appendix in most of the female patients. These negative appendectomy cases were diagnosed post-operatively with gynaecological conditions such as pelvic inflammatory disease, ruptured corpus luteal cyst, and right ovarian cyst. Thus, it can be concluded that female patients tend to get a negative appendicectomy finding (17–19), given that the diagnosis of appendicitis is not easy in female patients as they tend to have other gynaecological conditions that mimic the signs and symptoms of appendicitis (12, 20).

The present study showed that the majority of patients who underwent appendicectomy where Malays in all three groups. There was a significant difference in ethnic proportion between Group 2 and Group 3. This is because the number of non-Malays in Group 3 was higher compared to other groups. However, our results could be biased because non-Malays tend to seek treatment at a private hospital rather than government hospital since most of them have an insurance policy, which would make our result a less accurate representation of the race distribution. A previous study (4) conducted in University Hospital (currently named as University Malaya Medical Centre, Malaysia) revealed that the proportion of race diagnosed with appendicitis, either acute or perforated, was similar between Malays and non-Malays.

The median duration of admission for Group 3 was the highest, i.e., five days, compared to only three days in Groups 1 and 2. Statistically, there was a significant difference in the duration of admission between the groups. Group 3 showed the highest median duration of admission since the subjects in that group presented with complicated appendicitis such as perforated appendicitis, sepsis and generalised peritonitis and needed to undergo a major surgery such as lower midline laparotomy. Due to the severity of the disease, some of the subjects had a slow recovery post-operatively, which required a prolonged period of admission. These results are consistent with a study by Towfigh et al. (21) who showed that the patients diagnosed with perforated appendicitis had more days of admission (about five days) duration compared to acute appendicitis (two days duration).

There was a significant difference in mean of TWBC, lymphocytes, and neutrophils counts between the groups. Group 3 showed the highest mean of TWBC and neutrophils count, while Group 1 showed the highest mean of lymphocytes count. As expected, subjects in Group 3 presented with high TWBC and neutrophils count as they had a more severe form of appendicitis. On the contrary, they had low lymphocytes count, which contributed to high NLRs. Mean of platelet count also showed a significant difference between the groups; Group 3 showed the lowest mean of platelet count followed by Group 2 and Group 1. This is because platelet count reduced in sepsis or severe forms of inflammation such as perforated appendicitis due to an increase in sequestration and destruction of large and activated platelets at sites of inflammation (22, 23).

This study demonstrated that NLR could be a useful diagnostic tool or adjunct in diagnosing acute appendicitis. Patients with a normal appendix or negative appendicectomy (Group 1) had a low NLR compared to acute appendicitis (Group 2) and perforated appendicitis patients (Group 3). This indicated a highly statistically significant difference in median NLR between the groups. Subjects of Groups 2 and 3 showed high NLR values due to low lymphocytes count and high neutrophils count. However, Group 1 showed normal count of both lymphocytes and neutrophils. It showed that NLR increased in appendicitis and further increases as the inflammation progresses. This result is consistent with the findings of previous studies (12, 24, 25). Although TWBC showed a significant association with appendicitis, NLR proved superior and has greater diagnostic accuracy compared to TWBC alone (16, 26, 27). In addition, NLR might be more sensitive than the TWBC for the diagnosis of appendicitis, as neutrophils are increased and lymphocytes are decreased by infectious diseases such as appendicitis (28). During inflammatory responses, the ratio of the leucocytes in circulatory system changes, which relative lymphopenia accompanies the increase of neutrophils occur as a result of physiological response to the stress condition, that makes the ratio of these two subgroups to each other can be used as the reagent of inflammation.

Perforated appendicitis group showed a significantly higher NLR value compared to acute appendicitis group in our study. This means that NLR can be used to differentiate between acute appendicitis and perforated appendicitis since NLR in perforated appendicitis is significantly elevated. As reported by Mehmet et al. (29), NLR value is a valuable marker in the diagnosis of acute appendicitis diagnosis and prediction of perforated appendicitis.

The cut-off point of NLR in this study showed a highly statistically significant difference in an intergroup comparison. It showed that NLR with a cut-off point of ≥ 3.11 could significantly differentiate normal appendix (Group 1) and acute appendicitis patients (Group 2), with a sensitivity of 75.2% and specificity of 68.7%, in diagnosing acute appendicitis. The cut-off point of NLR in Group 1 versus Group 3 was higher compared to Group 1 versus Group 2 with a value of ≥ 5.11. However, its sensitivity and specificity improved to 83.3% and 91.30%, respectively. The former cut-off point of NLR is lower than the numbers reported in previous studies such as 4.68 (6), 5.96 (12) and 3.91 (30). This is because the cut-off point of NLR in the present study was only based on the NLR values of normal appendix versus acute appendicitis, compared to the previous studies in which the cut-off point of NLR was based on the NLR values of normal appendix versus acute appendicitis and perforated appendicitis considered together. Therefore, this resulted in the higher NLR value for the positive appendicectomy group and the cut-off point value. Thus, the cut-off of point of NLR in the present study was more specific for the diagnosis of acute appendicitis. Meanwhile, the cut-off point of NLR for perforated appendicitis was ≥ 6.17, which is lower compared to previous reports (16, 30, 31) but higher than the cut-off point reported by Kahramanca et al. (12), with sensitivity of 76.32% and specificity of 58.72%. This indicates that NLR could also be beneficial in identifying perforated appendicitis from acute appendicitis patients. The results of the present study showed that NLR could be a reliable tool or adjunct in diagnosing acute appendicitis since it has good sensitivity and specificity compared to other parameters such as TWBC (sensitivity: 62%, specificity: 75%), CRP (sensitivity: 57%, specificity: 87%), mean platelet volume (sensitivity: 59%, specificity: 59.5%) and pro-calcitonin (sensitivity: 33%, specificity: 89%) (22, 32).

NLR also showed a substantial correlation with severity of appendicitis, where higher NLR was associated with a more severe form of appendicitis. In a study by Shimizu et al. (26), NLR values > 5 were found to be indicative of severe appendicitis. In addition, NLR also showed a significant correlation with the duration of admission in hospital with the severity of appendicitis. Higher NLR was positively associated with longer duration of admission or length of stay in the hospital. This study showed that patients with perforated appendicitis have a median of 2 extra in-patient days compared to acute appendicitis patients. As reported by Kelly et al. (30), NLR could be used to predict the length of stay of appendicitis patients, and it could be useful in the planning of discharge strategies for the patients. In other words, NLR could be used as a tool to assist in the diagnosis of acute appendicitis and can also be used as a predictor of severity of appendicitis and length of stay of patients in hospital care.

There are several limitations to our study stemming from its retrospective nature. It is difficult to completely ascertain if there are unknown confounding variables that affect NLR validity. In addition, only patients who underwent appendicectomy were analysed. Patients initially suspected with appendicitis but did not undergo appendicitis were not included in this study. It can be argued that these patients should be included in the analysis as they may have a different pathology but presented with right iliac fossa pain.

Indeed, NLR is a simple and readily available low-cost test, but randomised studies in larger patient cohort are required to determine if a standardised cut-off value for NLR can successfully diagnose acute appendicitis. Furthermore, it could be better to include other parameters such as CRP in the study to compare with NLR in order to prove NLR is superior to other available parameters.

## Conclusion

NLR with a sensitivity of 75.23% and specificity of 68.7% is a useful and reliable adjunct in diagnosing acute appendicitis. It can well differentiate the normal appendix from acute appendicitis; hence, it will help in reducing the rates of negative appendicectomy and its associated complications. It also could be used to predict the severity of appendicitis and the duration of hospital admission, following post-surgical recovery.

## Figures and Tables

**Table 1:** Sociodemographic and clinical profiles of the study population

Variable	Group 1 (*N* = 115)	Group 2 (*N* = 109)	Group 3 (*N* = 114)	*P*-value
Age				
Mean (SD)	26.61 (12.27)	24.71 (12.86)	26.36 (12.91)	0.479^1^
Median (IQR)	23.00 (16.00)	20.00 (18.00)	22.00 (15.00)	
Range	13.00–73.00	13.00–69.00	13.00–70.00	
Gender				
Male	28 (24.3%)	50 (45.9%)	83 (72.8%)	Refer Table 2
Female	87 (75.7%)	59 (54.1%)	31 (27.2%)	
Race				
Malay	98 (85.2%)	99 (90.8%)	89 (78.1%)	Refer Table 3
Non-Malay	17 (14.8%)	10 (9.2%)	25 (21.9%)	
Duration of admission				
Mean (SD)	3.50 (0.86)	3.58 (1.14)	5.04 (2.37)	
Median (IQR)^*^	3.00 (1.00)	3.00 (1.00)	5.00 (2.00)	< 0.001^3^
Range	2.00–7.00	2.00–8.00	2.00–21.00	
TWBC (10×9/L)				
Mean (SD)	9.73 (3.34)	12.98 (4.33)	16.20 (4.67)	< 0.001^1^
Median (IQR)	9.00 (3.00)	12.60 (6.30)	16.30 (5.40)	
Range	4.10–23.20	4.90–23.90	4.50–27.50	
Lymphocytes (10×3/uL)				
Mean (SD)	2.55 (1.13)	2.04 (0.91)	1.64 (0.84)	< 0.001^1^
Median (IQR)	2.40 (1.10)	1.90 (1.30)	1.45 (0.82)	
Range	0.60–8.70	0.50–5.60	0.40–4.50	
Neutrophils				
Mean (SD)	6.47 (2.76)	10.34 (4.48)	13.64 (4.74)	< 0.001^1^
Median (IQR)	5.80 (3.10)	9.80 (6.90)	13.30 (5.83)	
Range	1.50–17.50	2.30–21.50	3.70–25.30	
Platelets (10×9/L)				
Mean (SD)	308.14 (105.08)	306.98 (78.81)	282.39 (70.29)	0.041^1^
Median (IQR)	291.00 (121.00)	303.00 (88.00)	284.50 (99.00)	
Range	101.00–918.00	122.00–534.00	102.00–465.00	

Notes: SD = Standard deviation; IQR = Interquartile range

Group 1 (G1): normal appendix; Group 2 (G2): acute appendicitis; Group 3 (G3): perforated appendicitis

1 One way ANOVA test was applied

2 Pearson’s chi-squared test were applied

3 Kruskal-Wallis test was applied

**Table 2:** Comparisons of gender proportion between groups

Comparison	*X*^2^	*P*-value*
Group 1 versus Group 2	11.422 (1)	0.001
Group 2 versus Group 3	16.795 (1)	< 0.001
Group 1 versus Group3	53.825 (1)	< 0.001

* Chi-squared test

**Table 3:** Comparisons of race proportion between groups

Comparison	*X*^2^	*P*-value*
Group 1 versus Group 2	1.660 (1)	0.198
Group 2 versus Group 3	6.852 (1)	0.009
Group 1 versus Group3	1.953 (1)	0.162

* Chi-squared test

**Table 4:** Comparisons of NLR between groups

Variable	Group 1 (*N* = 115)	Group 2 (*N* = 109)	Group 3 (*N* = 114)	*P*-value
NLR				
Mean (SD)	2.95 (1.86)	6.66 (5.02)	10.66 (7.73)	
Median (IQR)	2.37 (1.84)	5.25 (6.81)	9.27 (7.07)	< 0.001
Range	0.53–11.66	0.82–23.60	0.91–63.25	

Notes: Group 1 (G1): normal appendix; Group 2 (G2): acute appendicitis; Group 3 (G3): perforated appendicitis

Kruskal-Wallis test with Bonferroni adjusted multiple post-hoc comparison was applied

Group 1 versus Group 2: *P* < 0.001; Group 2 versus Group 3: *P* < 0.001; Group 1 versus Group 3: *P* < 0.001

**Table 5:** Area under the receiver operating characteristic curves (AUC), sensitive and specificity of NLR in predicting the diagnosis of acute appendicitis and perforated appendicitis

Comparison	Cut-off point	AUC (95% CI)	*P*-value^1^	SN	SP	LR+	LR−	Correctly classified
Group 3 versus Group 1	≥ 5.11	0.93 (0.89,0.96)	< 0.001	83.33	91.30	9.58	0.18	87.34%
Group 2 versus Group 1	≥ 3.11	0.76 (0.69,0.82)	< 0.001	75.23	68.70	2.40	0.36	71.88%
Group 3 versus Group 2	≥ 6.17	0.70 (0.63,077)	< 0.001	76.32	58.72	1.85	0.40	67.71%

Notes: CI = confidence interval; Group 1: normal appendix; Group 2: acute appendicitis; Group 3: perforated appendicitis

1 Compare with null hypothesis AUC = 0.5

**Table 6:** Correlation of NLR with disease severity and duration of admission

	*R*	*P*-value*
NLR and severity	0.620	< 0.001
NLR and duration of admission	0.27	< 0.001

* Spearman correlation tests were applied
